# Insanitary Surroundings

**Published:** 1916-08

**Authors:** 


### EDITORIAL DEPARTMENT

MRS. F. E. DANIEL, Publisher and Managing Editor.

### Insanitary Surroundings.

When the insanitary conditions in most cities and towns and about most country homes are considered there is not much occasion for surprise at the many epidemics that are constantly taking their immense toll of life in this country. The press of the country, and especially the medical press, has been preaching, teaching and pleading for better conditions for years, and some improvement has been made, but it is so little that it is hardly perceptible. There are in every city in the land open closets that are so filthy that they discredit our claims of being a refined or civilized people. Right in Austin, the Capital of the State, where the journal is edited, and under the very nostrils of the State authorities, are filthy, germ-laden, disease-breeding places enough to make the people wonder whether there is any serious earnestness in the efforts to inculcate the practice of sanitation as a means of improving the public health. Running right through the heart of this beautiful city, close by the Capitol, are half-stagnant streams that are but open sewers for the hundreds of closets near their banks, and for the offal and refuse of the homes and stables that drain into them. Within a block of the Capitol and right by fashionable churches, homes for working people, restaurants, and places where thousands work, eat or sleep are unscreened horse barns in front of at least one of which almost constantly stands, right on the public street, a wagon loaded with decaying stable manure. Alleys reek with the vile smells of privies and hog pens. In all these places flies breed, feed, and scatter at will over the city to carry their filth into the homes and into the food. Unscreend fruit and vegetable stands and wagons—favorite lounging places for the flies when they leave the breeding places—supply the people, poor and rich alike, with their food. Is there any wonder that people sicken and die under such conditions?

Austin has been mentioned because it is the home of the writer, and because it is the headquarters of the State Health Department, and is regarded as one of the healthiest cities in the country. It is no worse than any other city in the country, perhaps, and in some respects not so bad. Nevertheless, it is a shame that any city, especially a State Capital, should be so neglectful of its health conditions. It is little short of criminal that more concerted effort is not made to keep our towns and cities free from conditions such as have been mentioned.

Those who are in charge of the public health of a city are in large measure responsible where epidemics take place that could be prevented. We look upon war as cruel and barbarous, which it is. The little children who are dying by the hundreds in this country from infantile paralysis—the latest scourge due to official neglect and public indifference—are murdered even more inhumanely than the men who stand blindfolded before the firing squads of an enemy. Will the physicians stand idly by, and by their silence approve a continuation of conditions which they know produce nine-tenths of the epidemic sickness and deaths of the country? In the face of their knowledge of the causes that create such things, can they keep clear consciences without raising their voices unitedly in protest?

Officials become indifferent unless they are prodded to do their duty. The people generally are careless as to public health unless brought face to face with some appalling contagion, and even then they soon forget. Millions are so ignorant that they do not know anything about the causes that contribute to such things or how to stop them. The physicians are the volunteer guardians of the health of the people. It is no longer merely their duty to heal, but it is equally their obligation to prevent disease. How long will they, either as individuals or as organizations, neglect this self-assumed duty? Every physician in the land should get behind the State, county and city officials in such a way as to impel better conditions. They should talk to the people about them wherever they go, should instruct in sanitary improvement, should fill the press with warnings of the dangers, should personally entreat officials to be alert in conserving the health of the people, and point out the ways and means of bringing about improvement.

